# Factors Associated with 30-Day Non-Return to Pre-Hospital Residence in Older Patients Hospitalized with Pneumonia or Lower Respiratory Tract Infection: The Role of Serum Albumin and Early Treatment Failure

**DOI:** 10.3390/geriatrics11040101

**Published:** 2026-08-10

**Authors:** Akira Goto, Takayoshi Hozuki, Akihiro Matsumura, Hiroaki Mita, Norikazu Iwata, Emiko Tsuda, Ikumi Ebisu, Kazumi Yonekura, Susumu Kikuchi, Masahiro Nakamura, Yukinari Yoshida, Yuji Hinoda, Hiroo Yuasa, Yasuo Kato

**Affiliations:** 1 Department of Gastroenterology, Sapporo Shirakabadai Hospital, 7-26, Tsukisamu Higashi 2-jo 18-chome, Toyohira-ku, Sapporo 062-0052, Japan; 2 Department of Neurology, Sapporo Shirakabadai Hospital, Sapporo 062-0052, Japan; 3 Department of Cardiology, Sapporo Shirakabadai Hospital, Sapporo 062-0052, Japan

**Keywords:** pneumonia, lower respiratory tract infection, older adults, serum albumin, initial treatment failure, residential return, patient-centered outcome, community hospital

## Abstract

**Background/Objectives:** Return to the pre-hospital residence is a meaningful patient-centered outcome after hospitalization for pneumonia or lower respiratory tract infection (LRTI). We examined factors associated with 30-day non-return, including prespecified baseline characteristics, the initial empirical antimicrobial therapy pattern, and initial treatment failure within 7 days. **Methods:** This retrospective observational study included 126 consecutive patients aged ≥65 years admitted with pneumonia or LRTI to a community-based general hospital in Japan. Patients who died within 48 h were excluded. Three multivariable logistic regression models were fitted: Model 1 evaluated prespecified baseline characteristics; Models 2 and 3 added the initial empirical antimicrobial therapy pattern and initial treatment failure within 7 days, respectively. Initial treatment failure was treated as a post-baseline marker of an unfavorable early clinical course. **Results:** The median age was 89.0 years, and 56 patients (44.4%) returned to their pre-hospital residence within 30 days. Higher serum albumin was associated with lower adjusted odds of non-return across all models (OR range, 0.365–0.407 per 1 g/dL increase). Initial treatment failure was associated with higher adjusted odds of non-return (OR, 3.296; 95% CI, 1.095–9.923), whereas the initial empirical antimicrobial therapy pattern showed no statistically significant overall association (global *p* = 0.859). The principal findings were materially unchanged in sensitivity analyses addressing early deaths and the treatment-failure definition. However, in other supportive analyses, the associations remained directionally consistent but were attenuated and no longer statistically significant in the analysis restricted to 30-day survivors; in addition, the serum albumin association in Model 3 did not retain statistical significance in some leave-one-out analyses. **Conclusions:** Higher serum albumin at admission was associated with lower odds of 30-day non-return, whereas initial treatment failure was associated with higher odds. Initial treatment failure should be interpreted as a post-baseline marker of an unfavorable early clinical course rather than as an independent baseline predictor. No statistically significant association with the initial empirical antimicrobial therapy pattern was detected. These findings do not establish causality.

## 1. Introduction

Japan has one of the most rapidly aging populations in the world [1], and the increasing number of emergency admissions among older adults places a substantial burden on the healthcare system [2]. Pneumonia is a leading cause of hospitalization in this population and contributes significantly to the national healthcare burden [3,4]. However, tertiary acute-care hospitals face growing challenges in accommodating all older patients with pneumonia because of limited healthcare resources and physician shortages [5]. Consequently, community-based general hospitals have become essential providers of geriatric care and important backup institutions for home medical care and long-term care facilities [1,6].

In older adults, the goal of pneumonia management extends beyond resolution of the infection itself. Pneumonia frequently causes declines in activities of daily living (ADL) and prolonged hospitalization, hindering return to the pre-hospital living environment [7,8,9,10]. Therefore, return to the pre-hospital residence—whether home or a long-term care facility—represents an important patient-centered outcome [9,11].

To address these complex care needs, our community-based integrated-care general hospital accepts older patients with acute medical illnesses from both home and long-term care facilities. Despite the absence of respiratory specialists at our institution, internists from various specialties collaboratively manage these patients in routine clinical practice. Furthermore, our hospital integrates acute, rehabilitation, and chronic-care wards, facilitating seamless and continuous treatment and rehabilitation until discharge.

However, limited evidence exists regarding the factors associated with return to the pre-hospital living environment within such integrated community-based care systems, particularly among older patients. In addition, although guideline-adherent antimicrobial therapy is generally recommended for pneumonia management, its relationship with patient-centered outcomes such as residential return remains unclear in older adults. Beyond the initial antimicrobial choice, the early clinical response may also be relevant to subsequent recovery. Initial treatment failure within 7 days may serve as a marker of an unfavorable early clinical course because an inadequate response or early clinical deterioration may be associated with prolonged acute illness, delayed functional recovery, and a lower likelihood of return. Therefore, this study aimed to investigate factors associated with non-return to the pre-hospital residence within 30 days among older patients hospitalized with pneumonia or lower respiratory tract infection (LRTI) at our community-based integrated-care hospital.

## 2. Methods

### 2.1. Study Design and Setting

This retrospective observational study was conducted at a single-center, community-based integrated-care general hospital in Japan. The study patients were managed by 13 internists, including six gastroenterologists, six neurologists, and one cardiologist. No pulmonologists participated in their care.

### 2.2. Patients and Eligibility Criteria

**Figure 1:**
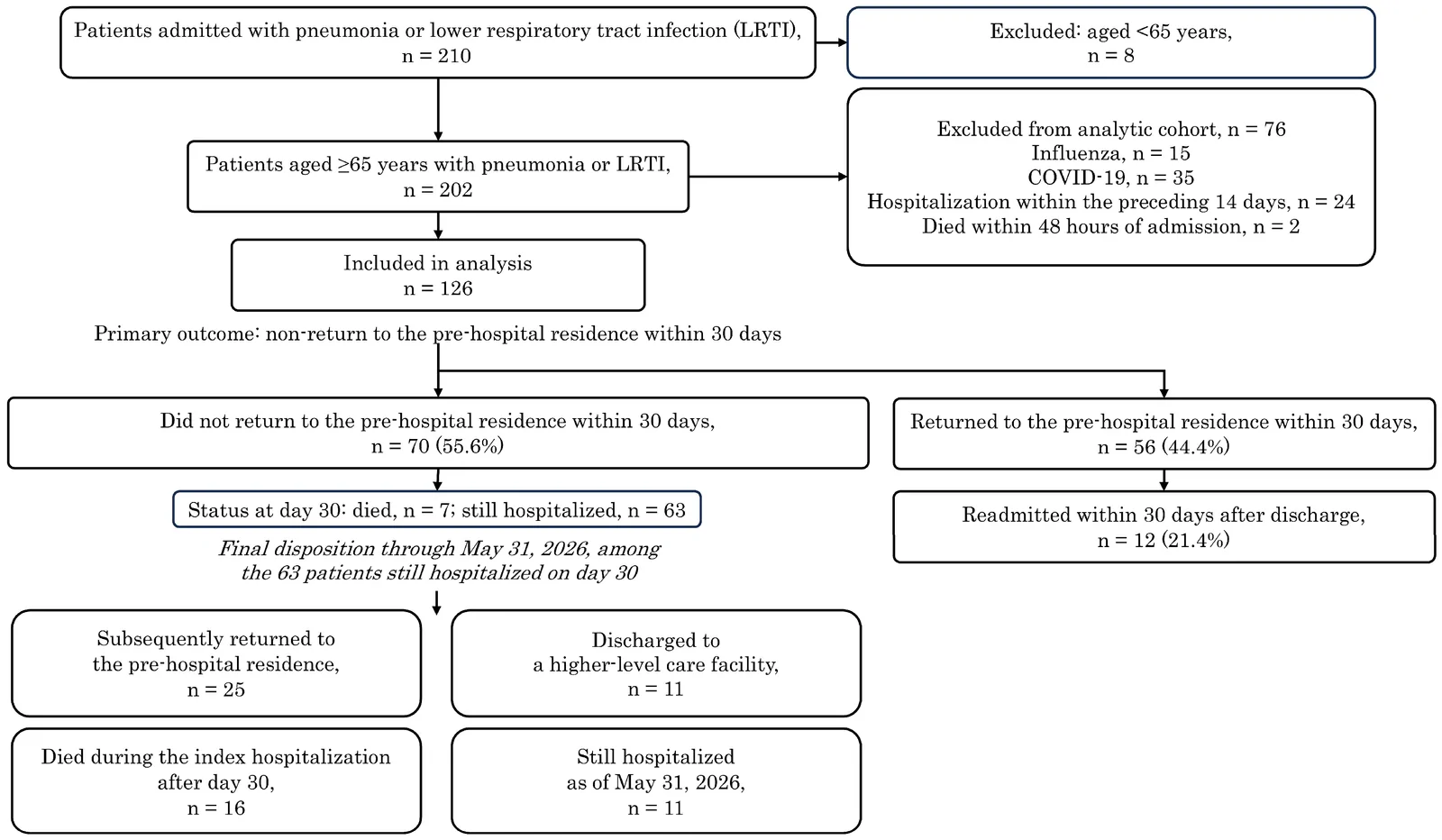
Patient flow diagram. LRTI, lower respiratory tract infection.

We retrospectively included consecutive patients aged 65 years or older who were admitted between 1 October 2024 and 31 March 2026, with pneumonia or LRTI. Patients younger than 65 years and those meeting the exclusion criteria described below were excluded. The patient selection process is shown in Figure 1.

Eligibility required clinical features suggestive of LRTI, such as fever, cough, or increased sputum production; no other evident source of infection; and initiation of empirical antimicrobial therapy for clinically suspected pneumonia or LRTI. Diagnosis was based on the attending physician’s overall assessment of clinical, laboratory, and imaging findings rather than on imaging findings alone. Chest computed tomography was performed in all patients as part of the initial evaluation, and no patient without chest imaging was included. However, pulmonary abnormalities on initial imaging may be subtle or nonspecific early in the clinical course. Particularly in older adults, findings suggestive of pneumonia may also be difficult to distinguish from pre-existing pulmonary abnormalities or noninfectious conditions [12,13]. Therefore, patients with clinically suspected LRTI were included even when imaging findings, including subtle or minor abnormalities, could not be clearly attributed to a new infectious process rather than a pre-existing or noninfectious condition.

Patients were excluded if they had been hospitalized within 14 days before the current admission, had tested positive for SARS-CoV-2 or influenza within 14 days of admission, or died within 48 h after admission. Patients who died within 48 h were excluded because their outcomes were considered to predominantly reflect extreme severity at presentation rather than the subsequent in-hospital clinical course.

### 2.3. Outcomes and Definitions

The primary outcome was non-return to the pre-hospital residence within 30 days of admission. Return was defined as discharge to the same residence as before admission, whether home or the same long-term care facility. Subsequent readmission did not alter this classification. Non-return was defined as death before return, discharge or transfer to a destination other than the pre-hospital residence, or continued hospitalization on day 30. Transfers to rehabilitation or chronic-care wards within the same hospital were considered continuation of the index hospitalization. This composite classification was used as an operational measure of whether patients achieved an initial return to the pre-hospital residence within 30 days. The components of non-return were not clinically equivalent. Accordingly, this outcome represents initial return within 30 days rather than sustained residence at the pre-hospital location.

The observation period for the primary outcome was 30 days from admission. For patients who returned to the pre-hospital residence, readmission was assessed for 30 days after discharge. For patients who remained hospitalized on day 30, subsequent disposition was descriptively assessed through 31 May 2026.

Initial treatment failure within 7 days after admission was defined as an unplanned change or escalation of antimicrobial therapy, or other treatment intensification, because of an inadequate clinical response or deterioration documented in the medical record. Treatment intensification included initiation of supplemental oxygen, an increase in oxygen support beyond the initial level, or initiation of vasopressor therapy. Treatment failure was supported by documented clinical findings such as persistent fever, persistently elevated or worsening inflammatory markers, increasing oxygen requirements, circulatory instability, or deterioration in mental status. All-cause in-hospital death within 7 days was also classified as treatment failure.

Pneumonia severity at admission was assessed using the A-DROP scoring system, which assigns one point each for age (men aged ≥70 years or women aged ≥75 years), dehydration (blood urea nitrogen ≥ 21 mg/dL or clinical dehydration), respiratory failure (SpO_2_ ≤ 90% or PaO_2_ ≤ 60 mm Hg), orientation disturbance, and low blood pressure (systolic blood pressure ≤ 90 mm Hg) [14,15]. Total scores of 0, 1–2, 3, and 4–5 were classified as mild, moderate, severe, and very severe, respectively; patients with shock were classified as very severe regardless of the total score. For subsequent classification and analysis, severity was dichotomized as mild/moderate versus severe/very severe.

Based on this severity classification, drug-resistant pathogen risk was assessed according to the JRS Clinical Practice Guidelines for Pneumonia in Adults 2024 [14]. Drug-resistant pathogen risk was considered present when at least one of seven risk factors was identified in patients with severe/very severe pneumonia or when at least three were identified in those with mild/moderate pneumonia. The seven factors were early intubation with invasive mechanical ventilation after admission, antimicrobial use within the previous 90 days, enteral nutrition, hypoalbuminemia, immunosuppression, hospitalization within the previous 90 days, and detection of a drug-resistant pathogen within the previous year.

Using the pneumonia severity and drug-resistant pathogen risk determined above, initial empirical antimicrobial therapy was classified into four mutually exclusive categories with reference to the JRS Clinical Practice Guidelines for Pneumonia in Adults 2024 [14]: (1) guideline-adherent therapy, (2) non-adherence due to overtreatment, defined as the use of broader-spectrum antimicrobial therapy than recommended by the guideline in patients without drug-resistant pathogen risk factors, (3) non-adherence due to undertreatment, defined as insufficient antimicrobial coverage in patients with drug-resistant pathogen risk factors, and (4) antimicrobial regimens not specifically listed in the guidelines. Examples of regimens not specifically listed in the guidelines included piperacillin, sulbactam/cefoperazone, and cefmetazole.

### 2.4. Data Collection

Baseline clinical data were collected from the medical records, including age, sex, pre-hospital residence in a long-term care facility, gastrostomy status, body mass index, Charlson Comorbidity Index [16], diabetes mellitus, pre-hospital cognitive impairment, pre-hospital ADL, serum albumin and estimated glomerular filtration rate (eGFR), both measured at admission, pneumonia severity assessed using the A-DROP scoring system [14,15], and drug-resistant pathogen risk. We also collected data on whether patients received rehabilitation or nutrition support team (NST) intervention during hospitalization.

Pre-hospital cognitive impairment was considered present when the medical records documented dementia, use of anti-dementia medication, or chronic cognitive decline affecting ADL, based on information from family members, caregivers, or attending physicians. Pre-hospital ADL was categorized as independent or requiring partial assistance versus requiring total assistance. Renal function was categorized according to eGFR as <30, 30–59, or ≥60 mL/min/1.73 m^2^.

Rehabilitation and NST interventions were recorded as binary variables indicating whether each intervention was provided during hospitalization. Their timing, frequency, intensity, content, and duration were not systematically collected.

### 2.5. Statistical Analysis

No formal sample size calculation was performed because the sample size was determined by the number of eligible consecutive patients admitted during the prespecified study period. No data were missing for the variables included in the analyses; therefore, no imputation was performed.

The distributions of continuous variables were assessed using visual inspection of histograms and the Shapiro–Wilk test. Serum albumin was approximately normally distributed and was summarized as the mean and standard deviation and compared using Student’s *t* test. Age, body mass index, and the Charlson Comorbidity Index were not considered approximately normally distributed and were summarized as medians and interquartile ranges and compared using the Mann–Whitney U test. Categorical variables were compared using Fisher’s exact test for 2 × 2 tables and the Fisher–Freeman–Halton exact test for contingency tables with more than two categories.

Multivariable logistic regression was used to evaluate adjusted associations with non-return to the pre-hospital residence within 30 days, which was coded as the outcome event. To limit model complexity relative to the sample size and number of outcome events, the baseline adjustment set was restricted to six variables prespecified on the basis of clinical relevance and prior knowledge rather than data-driven selection. These variables represented demographic characteristics, pre-hospital functional and cognitive status, nutritional and physiological reserve, and acute disease severity. Covariates were not selected using univariable *p* values, stepwise procedures, or other data-driven methods.

Model 1 included age, sex, pre-hospital activities of daily living, pre-hospital cognitive impairment, serum albumin, and A-DROP severity. Age and serum albumin were entered as continuous variables, with odds ratios expressed per 1-year and 1 g/dL increase, respectively. No categorical threshold for hypoalbuminemia was used in the primary regression models.

In-hospital rehabilitation and NST interventions were not included in the baseline adjustment set because they were post-baseline care processes that could be influenced by baseline characteristics and the early clinical course. They were therefore treated as descriptive variables rather than covariates in the primary multivariable models.

Model 2 included all Model 1 covariates plus the initial empirical antimicrobial therapy pattern. The four-category therapy-pattern variable was treated as nominal and entered using three indicator variables, with guideline-adherent therapy as the reference category. Its overall association with non-return was evaluated using a 3-degree-of-freedom likelihood-ratio test comparing Model 2 with Model 1. Model 3 included all Model 1 covariates plus initial treatment failure within 7 days after admission. Adjusted odds ratios and 95% confidence intervals were calculated for all three models (Table S1A–C).

Because death within 7 days contributed to both the definition of initial treatment failure and the composite primary outcome, initial treatment failure was treated in Model 3 as a post-baseline marker of an unfavorable early clinical course rather than as a causal exposure. Two sensitivity analyses specifically addressed this definitional overlap. Model 3 was repeated using a modified definition of initial treatment failure that excluded death as a qualifying criterion, and Model 3 was repeated after excluding the patient who died within 7 days (Tables S2A and S2B, respectively).

An additional sensitivity analysis repeated all three primary models after including the two patients who died within 48 h of admission, resulting in an analytic cohort of 128 patients (Table S3).

Model complexity was assessed by calculating the number of primary outcome events per estimated regression parameter, excluding the intercept. Because Model 2 had the lowest events-per-parameter ratio and some antimicrobial therapy categories contained relatively few patients, Model 2 was additionally fitted using Firth bias-reduced logistic regression. Confidence intervals and *p* values were based on profile penalized likelihood, and the three therapy-pattern indicator coefficients were tested jointly using a profile penalized likelihood-ratio test (Table S4).

Because age is a component of A-DROP, age was retained in the primary models as a potentially important independent confounder, and alternative models excluding either age or A-DROP were fitted to assess the influence of this structural overlap. Additional alternative-specification models adjusted for pre-hospital residence to assess potential confounding by baseline living setting (Table S5).

Drug-resistant pathogen risk was not included in the primary adjustment set because its classification partly depended on A-DROP severity and included a hypoalbuminemia criterion, resulting in structural overlap with the prespecified baseline covariates. In Model 2, drug-resistant pathogen risk was also used to define the initial empirical antimicrobial therapy pattern, creating additional definitional dependence. Exploratory models adding drug-resistant pathogen risk to Models 1 and 3 were fitted to assess whether its omission materially affected the principal estimates (Table S6). An interaction between drug-resistant pathogen risk and the four-category antimicrobial therapy pattern was not estimated because the antimicrobial therapy pattern was partly defined by the risk classification and several cross-classified groups were too small for reliable estimation.

Models 1 and 3 were also repeated in patients aged 80 years or older to assess whether the principal associations were similar in the oldest segment of the cohort. This analysis was considered supportive (Table S7).

An exploratory analysis restricted to patients who survived for 30 days was performed to assess the potential influence of deaths included in the composite primary outcome. Because conditioning on survival can introduce selection bias, this analysis was interpreted cautiously (Table S8A,B).

Multicollinearity was assessed using variance inflation factors, grouped generalized variance inflation factors, and condition indices. Influential observations were assessed using Cook’s distance, leverage, DFBETAS, and leave-one-out analyses of the principal estimates. No observations were excluded from the primary analyses on the basis of statistical influence (Table S9A,B). Discrimination and calibration were not formally assessed because the models were intended to estimate adjusted associations rather than individual-level predictive performance.

The linearity of age and serum albumin on the logit scale was assessed using restricted cubic splines with knots at the 10th, 50th, and 90th percentiles. Spline and linear specifications were compared using 1-degree-of-freedom likelihood-ratio tests (Table S10).

Primary analyses were performed using EZR version 1.70 (Saitama Medical Center, Jichi Medical University, Saitama, Japan), a graphical user interface for R (The R Foundation for Statistical Computing, Vienna, Austria) [17]. Additional analyses and regression diagnostics were performed using Python version 3.13.5 with statsmodels version 0.14.6 and SciPy version 1.17.0. All tests were two-sided, and *p* < 0.05 was considered statistically significant. *p* values were reported to three decimal places, with values less than 0.001 reported as *p* < 0.001. No adjustment for multiple comparisons was applied because the primary regression models addressed prespecified clinical questions and were interpreted separately. Sensitivity, subgroup, alternative-specification, and diagnostic analyses were considered supportive or exploratory and were interpreted cautiously.

## 3. Ethical Considerations

This study was conducted in accordance with the principles of the Declaration of Helsinki. The study protocol was approved by the Institutional Review Board of Sapporo Shirakabadai Hospital (Approval No. 2026003). Because this was a retrospective observational study using de-identified data, the requirement for individual informed consent was waived by the Institutional Review Board. Information about the study was made publicly available on the hospital website and through notices posted within the hospital, providing patients or their representatives with an opportunity to opt out.

## 4. Results

Among 210 patients admitted with pneumonia or LRTI during the study period, 8 patients younger than 65 years were excluded. Of the remaining 202 patients, 76 were excluded: 74 because of influenza, COVID-19, or hospitalization within the preceding 14 days, and 2 because they died within 48 h of admission. The final analytic cohort comprised 126 patients (Figure 1).

All 126 patients had some degree of radiographic pulmonary abnormality, ranging from subtle to overt, on chest CT. Because pneumonia and LRTI were not prospectively classified using uniform criteria, a reliable numerical breakdown between the two categories was not available.

Among the 126 patients, 56 (44.4%) returned to their pre-hospital residence within 30 days, whereas 70 (55.6%) did not. Of the 70 patients in the non-return group, 7 died within 30 days and 63 remained hospitalized on day 30. No patient was discharged or transferred to a destination other than the pre-hospital residence within 30 days.

Of the 63 patients who remained hospitalized on day 30, 25 subsequently returned to their pre-hospital residence, 16 died during the index hospitalization, 11 were discharged or transferred to a facility providing a higher level of care, and 11 remained hospitalized as of May 31, 2026. Overall, after excluding the 11 patients who remained hospitalized as of that date, index hospitalization outcomes were available for 115 patients, of whom 23 (20.0%) died during the index hospitalization.

Among the 56 patients who returned to their pre-hospital residence within 30 days, 12 (21.4%) were readmitted within 30 days after discharge.

Baseline clinical and treatment characteristics are shown in Table 1.

This was a predominantly very old cohort: the median age was 89.0 years (IQR, 84.0–93.8; range, 65–103 years), 107 patients (84.9%) were aged 80 years or older, and 93 (73.8%) were aged 85 years or older. Before admission, 72.2% of patients resided in long-term care facilities. Pre-hospital cognitive impairment was present in 78.6% of patients, and nearly half required total assistance with ADL.

In the unadjusted comparisons, pre-hospital cognitive impairment was more frequent in the non-return group than in the return group (85.7% vs. 69.6%, *p* = 0.048). The non-return group also had a lower mean serum albumin level (2.97 ± 0.52 vs. 3.24 ± 0.44 g/dL, *p* = 0.002) and a higher prevalence of drug-resistant pathogen risk (44.3% vs. 25.0%, *p* = 0.027). Pre-hospital total assistance with ADL (55.7% vs. 39.3%, *p* = 0.075) and severe or very severe disease according to A-DROP (40.0% vs. 23.2%, *p* = 0.056) were more frequent in the non-return group, although these differences did not reach statistical significance.

The initial empirical antimicrobial therapy pattern was classified as guideline-adherent therapy in 57 patients (45.2%), overtreatment in 14 (11.1%), undertreatment in 28 (22.2%), and a regimen not specifically listed in the guidelines in 27 (21.4%). The distribution of these four treatment patterns did not differ significantly between the return and non-return groups (*p* = 0.423). In contrast, initial treatment failure within 7 days was more frequent in the non-return group than in the return group (28.6% vs. 10.7%, *p* = 0.015).

Initial treatment failure occurred in 26 patients. Of these, 25 underwent an unplanned antimicrobial change or escalation within 7 days, and 1 patient died within 7 days without prior antimicrobial modification. Among these 25 patients, 8 also required initiation or intensification of oxygen support and 2 required vasopressor therapy; these components were not mutually exclusive.

Rehabilitation was provided to 98 patients (77.8%) and was more frequent in the non-return group than in the return group (87.1% vs. 66.1%, *p* = 0.005). NST intervention was provided to 39 patients (31.0%), with no significant difference between the groups.

The adjusted associations with non-return to the pre-hospital residence within 30 days are shown in Table 2.

There were no missing data for the outcome or covariates included in the primary models, and all 126 patients were included in Models 1–3. In Model 1, which included the six prespecified baseline covariates, a higher serum albumin level was associated with lower odds of non-return (OR, 0.377 per 1 g/dL increase; 95% CI, 0.162–0.879; *p* = 0.024). None of the other baseline covariates was significantly associated with the outcome after adjustment.

In Model 2, which added the four-category initial empirical antimicrobial therapy pattern, serum albumin remained associated with lower odds of non-return (OR, 0.365; 95% CI, 0.155–0.860; *p* = 0.021). The initial empirical antimicrobial therapy pattern showed no statistically significant association with the outcome as an overall four-category variable (3-degree-of-freedom likelihood-ratio test, *p* = 0.859). Compared with guideline-adherent therapy, no individual treatment-pattern category was significantly associated with non-return: overtreatment (OR, 0.788; 95% CI, 0.210–2.962; *p* = 0.725), undertreatment (OR, 0.718; 95% CI, 0.182–2.826; *p* = 0.635), and regimens not specifically listed in the guidelines (OR, 0.659; 95% CI, 0.239–1.814; *p* = 0.419).

In Model 3, which incorporated initial treatment failure as a post-baseline marker of an unfavorable early clinical course, serum albumin remained associated with lower adjusted odds of non-return (OR, 0.407; 95% CI, 0.170–0.974; *p* = 0.044). Initial treatment failure within 7 days was associated with higher adjusted odds of non-return (OR, 3.296; 95% CI, 1.095–9.923; *p* = 0.034).

In a sensitivity analysis using a modified definition of initial treatment failure that excluded death as a qualifying criterion, the results of Model 3 were materially unchanged. Serum albumin remained associated with lower odds of non-return (OR, 0.401; 95% CI, 0.168–0.958; *p* = 0.040), and modified initial treatment failure remained associated with higher odds of non-return (OR, 3.177; 95% CI, 1.045–9.663; *p* = 0.042) (Table S2A).

In a second sensitivity analysis excluding the patient who died within 7 days, the results of Model 3 were also materially unchanged. Serum albumin remained associated with lower odds of non-return (OR, 0.411; 95% CI, 0.172–0.982; *p* = 0.045), and initial treatment failure remained associated with higher odds of non-return (OR, 3.192; 95% CI, 1.052–9.683; *p* = 0.040) (Table S2B).

In a sensitivity analysis including the two patients who died within 48 h of admission (N = 128), the direction, magnitude, and statistical interpretation of the principal associations were materially unchanged. Serum albumin remained associated with lower odds of non-return in all three models, initial treatment failure remained associated with higher odds of non-return in Model 3, and no statistically significant overall association was detected for the initial empirical antimicrobial therapy pattern in Model 2 (Table S3).

The numbers of primary outcome events per estimated parameter were 11.7 for Model 1, 7.8 for Model 2, and 10.0 for Model 3. Because Model 2 had the lowest ratio, it was repeated using Firth bias-reduced logistic regression. The global association of the antimicrobial therapy pattern remained non-significant (profile penalized likelihood-ratio *p* = 0.883), and the coefficient estimates were similar to those from standard logistic regression (Table S4).

In alternative models excluding either age or A-DROP and in models additionally adjusting for pre-hospital residence, the directions and approximate magnitudes of the associations for serum albumin and initial treatment failure were materially unchanged (Table S5).

Because drug-resistant pathogen risk was not included in the primary adjustment set, its potential influence was examined in exploratory analyses. Drug-resistant pathogen risk showed substantial overlap with A-DROP severity: 38 of 41 patients (92.7%) with severe or very severe A-DROP were classified as having drug-resistant pathogen risk, compared with 7 of 85 patients (8.2%) with mild or moderate A-DROP. After additional adjustment for drug-resistant pathogen risk in Models 1 and 3, the risk variable itself was not statistically associated with non-return, and the principal estimates for serum albumin and initial treatment failure were materially similar to those in the corresponding primary models. In Model 3 with additional adjustment for drug-resistant pathogen risk, the ORs were 0.420 for serum albumin (95% CI, 0.173–1.023; *p* = 0.056) and 3.360 for initial treatment failure (95% CI, 1.107–10.194; *p* = 0.032) (Table S6).

A sensitivity analysis restricted to patients aged 80 years or older (N = 107) showed associations for serum albumin and initial treatment failure in the same direction and of similar magnitude to those in the full cohort (Table S7).

In the exploratory analysis restricted to 30-day survivors, the associations remained in the same direction but were attenuated and no longer statistically significant (Table S8A,B).

Multicollinearity diagnostics did not indicate substantial numerical instability: the maximum variance inflation factor was 2.260, the adjusted generalized variance inflation factor for the antimicrobial therapy pattern was 1.145, and the maximum condition index was 2.895. Influence diagnostics identified some observations exceeding conservative screening thresholds but no extreme influential observations. In leave-one-out analyses, the directions of the principal associations remained unchanged. The association of serum albumin remained statistically significant in all leave-one-out fits for Models 1 and 2, whereas its statistical significance was borderline in some fits for Model 3; the association with initial treatment failure was directionally and quantitatively stable (Table S9A,B).

Restricted cubic spline analyses showed no evidence of departure from linearity for serum albumin or age in Models 1 or 3 (all *p* for nonlinearity ≥ 0.204) (Table S10; Figures S1 and S2).

Full results of the sensitivity, alternative-specification, and diagnostic analyses are provided in the Supplementary Materials.

## 5. Discussion

This retrospective observational study examined 30-day non-return to the pre-hospital residence as a patient-centered outcome in a predominantly very old cohort hospitalized with pneumonia or LRTI at a community-based integrated-care general hospital. Fewer than half of the patients returned within 30 days. The principal findings were that lower serum albumin and initial treatment failure within 7 days were associated with 30-day non-return after multivariable adjustment, whereas the initial empirical antimicrobial therapy pattern was not significantly associated with this outcome. The directions of the associations for serum albumin and initial treatment failure were generally maintained across sensitivity and alternative-specification analyses. However, in the exploratory analysis restricted to 30-day survivors, both associations were attenuated and no longer statistically significant, and the statistical significance of the serum albumin estimate in Model 3 was not retained in some leave-one-out analyses. The principal estimates should therefore be interpreted as directionally consistent but not uniformly statistically robust. In older adults, particularly the oldest patients, survival alone may not represent successful recovery because acute illness and hospitalization can lead to functional decline and loss of independence; residential return may therefore provide clinically meaningful information beyond conventional clinical outcomes.

Lower serum albumin was consistently associated with a higher likelihood of 30-day non-return across the multivariable models. Serum albumin is a nonspecific marker reflecting nutritional status, inflammatory burden, underlying disease, and physiological reserve. In very old patients, lower serum albumin often accompanies reduced muscle mass, diminished physical reserve, and poorer recovery after acute illness [18,19]. Our finding is broadly consistent with a nationwide study of older patients with aspiration pneumonia, in which malnutrition, defined in part by a serum albumin level of 3.0 g/dL or lower, was associated with a lower likelihood of discharge to home or a nursing home [11]. Although that study was restricted to aspiration pneumonia and examined discharge destination rather than return to the pre-hospital residence, both findings suggest that serum albumin may serve as a marker of vulnerability relevant to recovery rather than merely as an isolated laboratory abnormality. However, serum albumin should not be interpreted as a specific measure of malnutrition or frailty, and the present observational findings do not establish that lower serum albumin directly causes 30-day non-return or that increasing serum albumin would improve the likelihood of return.

From a practical perspective, serum albumin is inexpensive, widely available, and routinely measured at hospital admission. Although nonspecific, a lower serum albumin level may serve as a simple signal to consider early multidisciplinary assessment, rehabilitation planning, nutritional evaluation, and coordinated discharge support.

Initial treatment failure within 7 days was associated with higher adjusted odds of 30-day non-return. This association persisted when death was removed from the definition of initial treatment failure and when the patient who died within 7 days was excluded, suggesting that the finding was not solely attributable to the overlap of early death between the exposure and outcome definitions. Initial treatment failure should be interpreted as a post-baseline marker of an unfavorable early clinical course rather than as a causal determinant or an independent baseline predictor of non-return. However, 25 of the 26 patients classified as having initial treatment failure underwent an unplanned antimicrobial modification or escalation. Therefore, this variable was predominantly based on a clinician-dependent management decision and may have captured differences in attending physicians’ thresholds for modifying therapy, in addition to biological deterioration or an inadequate treatment response. Although antimicrobial modification was generally prompted by documented clinical deterioration or an inadequate response, the timing and threshold for modification were not standardized or independently adjudicated. Accordingly, the observed association should not be interpreted as indicating that antimicrobial modification itself caused non-return. Nevertheless, because the definition also included documented clinical deterioration and escalation of respiratory or circulatory support, initial treatment failure may have reflected disease severity, treatment response, and patient vulnerability more broadly. Such an unfavorable early course may prolong acute illness, contribute to immobilization and functional decline, and delay recovery, thereby reducing the likelihood of return even when the infection is ultimately controlled [20]. Patients with initial treatment failure may therefore warrant closer monitoring and early reassessment.

Another notable finding was the relatively high 30-day readmission rate (21.4%) among patients who returned to their pre-hospital residence within 30 days. This finding suggests that clinical and functional vulnerability may have persisted after discharge. Previous studies have shown that readmissions after pneumonia are frequently related to comorbid conditions and are not limited to recurrent pneumonia [21,22]. These findings highlight the potential importance of post-discharge follow-up, coordination with home medical care services and long-term care facilities, and continued multidisciplinary support.

No statistically significant association between the initial empirical antimicrobial therapy pattern and 30-day non-return was detected in this cohort, consistent with the relative scarcity of prior studies examining this specific association in older adults; most previous work on antimicrobial strategies for pneumonia has instead focused on mortality, treatment success, or length of hospital stay. This null result should not be interpreted as demonstrating equivalence among empirical regimens or as indicating that antimicrobial selection is clinically unimportant. Several antimicrobial categories contained few patients, and the corresponding confidence intervals were wide, limiting the precision of category-specific estimates. In addition, antimicrobial selection may have been influenced by disease severity and perceived drug-resistant pathogen risk, and residual confounding by indication cannot be excluded.

The nature of the outcome may also help explain the lack of a statistically significant association. Initial antimicrobial selection is primarily intended to provide adequate treatment of the acute infection while avoiding unnecessary broad-spectrum exposure and the associated risks of adverse effects and antimicrobial resistance. In contrast, return to the pre-hospital residence within 30 days is influenced not only by infection control but also by baseline functional and cognitive status, nutritional and physiological reserve, recovery after acute illness, rehabilitation needs, family circumstances, and facility acceptance. Thus, potential effects of antimicrobial selection on microbiological or short-term infectious outcomes may not translate into a detectable association with this broader residential outcome in a modest cohort. Moreover, regimens categorized as “not specifically listed in the guidelines,” particularly sulbactam/cefoperazone and cefmetazole, may have been selected when aspiration was clinically suspected, and coverage of oral and anaerobic bacteria was considered appropriate. Given the advanced age of the cohort and the high prevalence of pre-hospital cognitive impairment, such clinical considerations may have influenced antimicrobial selection. Therefore, this classification should not be interpreted as indicating clinically inappropriate treatment.

Our hospital is a community-based integrated-care general hospital without an on-site pulmonologist, and patients were managed by internists from several subspecialty backgrounds. In Japan’s super-aged society, general internists have an increasingly important role in the routine management of older patients with pneumonia or LRTI, particularly in regions with limited specialist resources. Clinical practice guidelines provide a structured framework for severity assessment and empirical antimicrobial selection in this setting. Nevertheless, the absence of routine pulmonology involvement and variation among attending physicians may have influenced diagnostic classification, interpretation and application of guideline recommendations, antimicrobial selection, recognition of early treatment failure, and decisions regarding treatment modification or escalation. Because this study did not include a comparison group managed with routine pulmonology involvement, the effect of specialist absence on overall clinical outcomes could not be determined. These features should be considered when generalizing the findings to institutions with continuous respiratory specialist involvement.

Rehabilitation was more frequent in the non-return group, likely reflecting confounding by indication, because patients with greater functional impairment or clinical needs were more likely to receive it. NST intervention did not differ significantly between the return and non-return groups. Because the indications, timing, intensity, duration, and content of these interventions were not standardized or systematically collected, their effects on residential outcomes could not be determined in this study. These findings should therefore not be interpreted as indicating that rehabilitation or nutritional support was ineffective.

The association between lower serum albumin and 30-day non-return, together with the inability of the present study to evaluate the effects of nutritional intervention, provides a rationale for further investigation of nutritional vulnerability and targeted support during hospitalization. The EFFORT trial demonstrated that individualized nutritional support improved clinical and functional outcomes among hospitalized medical patients at nutritional risk [23]. The EFFORT findings suggest that targeted nutritional interventions merit evaluation in very old patients hospitalized with pneumonia or LRTI, although their applicability to this population and to return to the pre-hospital residence remains uncertain.

## 6. Limitations

Several limitations should be acknowledged. First, this retrospective study had a modest sample size, which limited statistical power and precision and restricted the number of covariates that could be included simultaneously. Although consecutive patients were included, selection bias cannot be excluded. Model 2 had the lowest number of outcome events per estimated parameter, and several category-specific estimates had wide confidence intervals. Firth regression, influence diagnostics, and leave-one-out analyses yielded generally consistent results; however, although the direction of the serum albumin association in Model 3 remained stable, statistical significance was not retained in some leave-one-out analyses. The models were therefore intended to estimate exploratory adjusted associations rather than to serve as validated prognostic models. They were not developed or validated for individual-level risk prediction, and discrimination, calibration, and predictive utility were not evaluated.

Second, the diagnosis of pneumonia or LRTI was based on the attending physician’s integrated clinical assessment rather than uniform prespecified diagnostic criteria. Although chest CT was performed in all patients, imaging findings could not always reliably distinguish new infectious infiltrates from pre-existing, residual, chronic, or noninfectious pulmonary abnormalities. The study hospital had no dedicated on-site pulmonologist, and no independent pulmonologist review or centralized radiological adjudication was performed. This may have affected diagnostic consistency and may also limit generalizability to institutions with routine respiratory specialist involvement. Aspiration pneumonia was not classified using uniform predefined criteria. Diagnostic heterogeneity and misclassification therefore remain possible. Because patients were not prospectively assigned to mutually exclusive pneumonia and LRTI categories using uniform criteria, a reliable numerical breakdown or subgroup analysis of pneumonia versus LRTI could not be provided.

Third, initial treatment failure was not an independently adjudicated biological outcome. Because 25 of the 26 patients meeting this definition underwent an unplanned antimicrobial modification or escalation, the classification was largely dependent on clinicians’ recognition of an inadequate response and their thresholds for modifying treatment. Although the medical records documented clinical findings supporting these decisions, the timing and threshold for treatment modification were not standardized, and inter-physician variation in assessment and management may have contributed to the classification. Accordingly, the observed association with non-return may reflect both the patient’s early clinical course and clinician-dependent management decisions.

Fourth, detailed geriatric assessments, including the Barthel Index, certified care-need level, frailty scales, swallowing function, and measures of sarcopenia, were not consistently available in the medical records. The binary classification of pre-hospital ADL was used because it was the most consistently documented functional measure across all patients. Patient and family preferences, facility acceptance, discharge coordination, and other social or institutional determinants of residential return were also unavailable. Although baseline covariates were prespecified on clinical grounds and the principal findings were generally consistent across alternative adjustment sets, residual and unmeasured confounding cannot be excluded. The reported estimates therefore represent adjusted associations within the measured covariate set and do not establish causal relationships.

Fifth, rehabilitation and NST interventions were recorded only as present or absent and were not standardized. Their indications, timing, frequency, intensity, content, and duration were not systematically collected. Because these post-baseline interventions may have been initiated in response to greater functional impairment, nutritional risk, disease severity, or clinical deterioration, their descriptive associations with residential outcomes are susceptible to confounding by indication. Accordingly, the observed differences cannot be taken as estimates of intervention effectiveness.

Sixth, the components of 30-day non-return differed in clinical severity and underlying mechanism and should not be regarded as equivalent outcomes. They were combined because each represented non-return to the pre-hospital residence within 30 days. Because death was included as one component, the observed associations may partly reflect associations with mortality. In the exploratory analysis restricted to 30-day survivors, the associations remained in the same direction but were attenuated and no longer statistically significant; this analysis had reduced power and may also have been affected by selection bias from conditioning on survival. In addition, return to the pre-hospital residence may not always be the optimal outcome, because transfer to a facility providing a higher level of support may be clinically appropriate for some patients. Because subsequent readmission did not alter the return classification, the outcome represented initial return within 30 days rather than sustained residence at the pre-hospital location. Thus, non-return should be interpreted as failure to achieve an initial return to the pre-hospital residence within 30 days rather than as an outcome that is uniformly unfavorable in every clinical context.

Seventh, the antimicrobial therapy classification was intrinsically related to drug-resistant pathogen risk because this risk contributed to the definitions of guideline-adherent therapy, overtreatment, and undertreatment. Exploratory adjustment for drug-resistant pathogen risk did not materially alter the principal estimates but could not fully separate antimicrobial spectrum from the clinical indications underlying treatment selection. Small numbers in several treatment categories and wide confidence intervals further limited the ability to detect moderate associations. The absence of a statistically significant association therefore does not demonstrate equivalence among antimicrobial strategies.

Eighth, serum albumin was measured only at admission and is a nonspecific marker influenced by nutritional status, systemic inflammation, liver function, hydration status, illness severity, and overall physiological reserve. Detailed longitudinal information on these factors, dietary intake, frailty, and body composition was unavailable. The serum albumin association may therefore reflect unmeasured aspects of patient vulnerability as well as nutritional status. These findings do not establish that lower serum albumin itself causes non-return or that interventions intended to increase serum albumin would improve residential outcomes.

Ninth, longer-term outcomes beyond the available follow-up, including long-term functional status, quality of life, and survival, were not evaluated.

## 7. Conclusions

In this single-center retrospective cohort of predominantly very old patients hospitalized with pneumonia or LRTI, lower admission serum albumin was associated with a higher likelihood of non-return to the pre-hospital residence within 30 days. Initial treatment failure, interpreted as a post-baseline marker of an unfavorable early clinical course, was also associated with higher odds of non-return. The directions of these associations were generally consistent across supportive analyses, although statistical significance was not uniformly retained, indicating limited precision and some sensitivity to analytical specification. No statistically significant association was detected between the initial empirical antimicrobial therapy pattern and this outcome. These findings should be interpreted as observational associations and do not establish causality, individual-level predictive performance, or equivalence among antimicrobial strategies.

## Figures and Tables

**Table 1:** Patient characteristics and treatment-related factors according to 30-day return status.

Variable	Total (n = 126)	Return (n = 56)	Non-Return (n = 70)	*p* Value
Age, years, median (IQR)	89 (84.0–93.8)	88 (82.7–93.0)	89 (87.0–94.0)	0.194 †
Male sex, n (%)	64 (50.8)	26 (46.4)	38 (54.3)	0.473 ‡
Pre-hospital residence in a long-term care facility, n (%)	91 (72.2)	40 (71.4)	51 (72.9)	1.000 ‡
Gastrostomy, n (%)	9 (7.1)	5 (8.9)	4 (5.7)	0.509 ‡
BMI, kg/m^2^, median (IQR)	19.2 (16.8–21.8)	19.7 (17.0–22.0)	18.6 (16.7–21.6)	0.288 †
Charlson Comorbidity Index, median (IQR)	2 (1–3)	2 (1–3)	2 (1–3)	0.127 †
Pre-hospital cognitive impairment, n (%)	99 (78.6)	39 (69.6)	60 (85.7)	0.048 ‡
Diabetes mellitus, n (%)	41 (32.5)	16 (28.6)	25 (35.7)	0.447 ‡
Pre-hospital ADL: total assistance, n (%)	61 (48.4)	22 (39.3)	39 (55.7)	0.075 ‡
Serum albumin, g/dL, mean ± SD	3.09 ± 0.50	3.24 ± 0.44	2.97 ± 0.52	0.002 §
eGFR, mL/min/1.73 m^2^, n (%)				0.659 ¶
<30	13 (10.3)	5 (8.9)	8 (11.4)	
30–59	58 (46.0)	24 (42.9)	34 (48.6)	
≥60	55 (43.7)	27 (48.2)	28 (40.0)	
A-DROP severity: severe/very severe, n (%)	41 (32.5)	13 (23.2)	28 (40.0)	0.056 ‡
Drug-resistant pathogen risk, n (%)	45 (35.7)	14 (25.0)	31 (44.3)	0.027 ‡
Initial empirical antimicrobial therapy pattern, n (%)				0.423 ¶
Guideline-adherent	57 (45.2)	26 (46.4)	31 (44.3)	
Overtreatment	14 (11.1)	8 (14.3)	6 (8.6)	
Undertreatment	28 (22.2)	9 (16.1)	19 (27.1)	
Regimen not specifically listed in the guidelines	27 (21.4)	13 (23.2)	14 (20.0)	
Initial treatment failure within 7 days, n (%)	26 (20.6)	6 (10.7)	20 (28.6)	0.015 ‡
In-hospital rehabilitation, n (%)	98 (77.8)	37 (66.1)	61 (87.1)	0.005 ‡
Nutrition support team intervention, n (%)	39 (31.0)	17 (30.4)	22 (31.4)	1.000 ‡

Abbreviations: ADL, activities of daily living; A-DROP, age, dehydration, respiratory failure, orientation disturbance, and blood pressure; BMI, body mass index; eGFR, estimated glomerular filtration rate; IQR, interquartile range; SD, standard deviation. † Mann–Whitney U test. ‡ Fisher’s exact test. § Student’s *t*-test. ¶ Fisher–Freeman–Halton exact test for r × c tables.

**Table 2:** Multivariable logistic regression analyses of factors associated with non-return to the pre-hospital residence within 30 days.

Variable	Model 1 (Main)		Model 2 (+ Initial Treatment Pattern)		Model 3 (+ Initial Treatment Failure)	
	OR (95% CI)	*p* Value	OR (95% CI)	*p* Value	OR (95% CI)	*p* Value
Age, per 1-year increase	1.023 (0.972–1.077)	0.388	1.023 (0.970–1.079)	0.407	1.029 (0.976–1.085)	0.287
Male sex (vs. female)	1.634 (0.744–3.586)	0.221	1.640 (0.744–3.617)	0.220	1.634 (0.732–3.648)	0.231
Pre-hospital ADL: total assistance (vs. independent/partial assistance)	1.358 (0.614–3.004)	0.449	1.416 (0.626–3.203)	0.404	1.463 (0.645–3.317)	0.363
Pre-hospital cognitive impairment (vs. no impairment)	2.043 (0.737–5.665)	0.170	2.116 (0.740–6.054)	0.162	2.162 (0.763–6.126)	0.147
Serum albumin, per 1 g/dL increase	0.377 (0.162–0.879)	0.024	0.365 (0.155–0.860)	0.021	0.407 (0.170–0.974)	0.044
A-DROP severity: severe/very severe (vs. mild/moderate)	1.920 (0.796–4.632)	0.147	2.174 (0.634–7.460)	0.217	1.609 (0.647–4.004)	0.306
Initial empirical antimicrobial therapy pattern, overall †	–		–	0.859	–	
Guideline-adherent			1.00 (Reference)			
Overtreatment			0.788 (0.210–2.962)	0.725		
Undertreatment			0.718 (0.182–2.826)	0.635		
Regimen not specifically listed in the guidelines			0.659 (0.239–1.814)	0.419		
Initial treatment failure within 7 days (vs. no failure)	–		–		3.296 (1.095–9.923)	0.034

Abbreviations: ADL, activities of daily living; A-DROP, age, dehydration, respiratory failure, orientation disturbance, and blood pressure; CI, confidence interval; OR, odds ratio. Model 1 was adjusted for age, sex, ADL status, cognitive impairment, serum albumin, and A-DROP severity. Model 2 additionally included the initial empirical antimicrobial therapy pattern, and Model 3 additionally included initial treatment failure within 7 days. Reference categories are indicated in the variable labels; guideline-adherent antimicrobial therapy was the reference category for the treatment-pattern variable. † The overall *p* value for the four-category initial empirical antimicrobial therapy pattern was calculated using a 3-degree-of-freedom likelihood-ratio test. Category-specific *p* values were calculated using Wald tests.

## Data Availability

The de-identified data analyzed in this study are not publicly available because of the small, single-center nature of the cohort and the risk of participant reidentification. De-identified data may be made available from the corresponding author upon reasonable request, subject to institutional approval.

## Supplementary Materials

The following supporting information can be downloaded at: https://www.mdpi.com/article/10.3390/geriatrics11040101/s1, Table S1A: Full standard logistic regression results: Model 1; Table S1B: Full standard logistic regression results: Model 2; Table S1C: Full standard logistic regression results: Model 3; Table S2A: Model 3 using a modified definition of initial treatment failure that excluded death as a qualifying criterion; Table S2B: Model 3 after excluding the patient who died within 7 days; Table S3: Sensitivity analysis including the two patients who died within 48 hours of admission (N = 128); Table S4: Firth bias-reduced logistic regression for Model 2; Table S5: Alternative adjustment sets; Table S6: Exploratory models with additional adjustment for drug-resistant pathogen risk; Table S7: Sensitivity analysis restricted to patients aged 80 years or older; Table S8A: Exploratory analysis restricted to 30-day survivors: Model 1; Table S8B: Exploratory analysis restricted to 30-day survivors: Model 3; Table S9A: Multicollinearity, influence, and model-complexity diagnostics; Table S9B: Leave-one-out robustness of key estimates; Table S10: Restricted cubic spline tests for nonlinearity; Figure S1: Restricted cubic spline assessment of serum albumin; Figure S2: Restricted cubic spline assessment of age.
