# Supplementary material for: Factors Associated with 30-Day Non-Return to Pre-Hospital Residence in Older Patients Hospitalized with Pneumonia or Lower Respiratory Tract Infection: The Role of Serum Albumin and Early Treatment Failure
Source: Geriatrics (Basel). 2026 Aug 10;11(4):101. doi: 10.3390/geriatrics11040101 (PMC13511903; doi:10.3390/geriatrics11040101)
Supplement: Supplementary file 1 [file geriatrics-11-00101-s001.zip › geriatrics-4412592-supplementary.pdf]

Supplementary Materials

Additional sensitivity analyses and regression diagnostics

Factors Associated with 30-Day Non-Return to Pre-Hospital Residence in Older Patients Hospitalized with Pneumonia or Lower Respiratory Tract Infection: The Role of Serum Albumin and Early Treatment Failure

This supplement reports additional analyses used to assess the robustness of the primary findings and the assumptions of the multivariable logistic regression models in the analytic cohort of 126 patients. Patients who died within 48 hours after admission were excluded according to the eligibility criteria of the submitted Geriatrics manuscript. These analyses were not used to select the primary model or redefine the study outcome. A sensitivity analysis repeating all primary models after including the two patients who died within 48 hours of admission, resulting in a cohort of 128 patients, is reported in Table S3.

Supplementary Methods

Standard logistic regression. The analytic cohort included 126 patients and 70 primary outcome events. Model 1 included age, sex, pre-hospital activities of daily living, pre-hospital cognitive impairment, serum albumin, and A-DROP severity. Model 2 added the four-category initial empirical antimicrobial therapy pattern, and Model 3 added initial treatment failure within 7 days. The overall contribution of the antimicrobial therapy pattern was evaluated using a 3-degree-of-freedom likelihood-ratio test.

Firth analysis. Model 2 was repeated using Firth bias-reduced logistic regression by maximizing the Jeffreys-prior penalized likelihood. Confidence intervals and P values were based on profile penalized likelihood. The antimicrobial therapy pattern was tested jointly by constraining its three indicator coefficients to zero while retaining the full-model penalty.

Linearity and diagnostics. The linearity of serum albumin and age on the logit scale was assessed using restricted cubic splines with knots at the 10th, 50th, and 90th percentiles. A 1-degree-of-freedom likelihood-ratio test compared spline and linear specifications. Multicollinearity was assessed using variance inflation factors, grouped generalized variance inflation factors, and condition indices. Influence was assessed using Cook's distance, leverage, and DFBETAS, followed by leave-one-out analyses.

Alternative and exploratory analyses. Because age is a component of A-DROP, alternative models excluding either age or A-DROP were fitted to assess the influence of their structural overlap. Additional models adjusted for pre-hospital residence to assess potential confounding by baseline living setting. Models 1 and 3 were also repeated in patients aged 80 years or older to assess the consistency of the principal associations within the oldest segment of the cohort. An exploratory analysis restricted to 30-day survivors was performed to assess whether the observed associations were driven by deaths included in the composite primary outcome. A further sensitivity analysis repeated the primary models after including the two patients who died within 48 hours of admission and were excluded from the primary analytic cohort under the study eligibility criteria; the rationale for this exclusion and the results of this analysis are reported in Table S3. To assess the potential overlap created by including death in both initial treatment failure and the primary outcome, Model 3 was additionally repeated using a modified definition of initial treatment failure that did not use death itself as a qualifying criterion. A complementary analysis excluded the patient who died within 7 days.

Full regression results

Table S1A. Full standard logistic regression results: Model 1

Variable	Coefficient β	SE	OR (95% CI)	P value
Intercept	0.088	2.633	1.092 (0.006–190.176)	0.973
Age, per 1-year increase	0.023	0.026	1.023 (0.972–1.077)	0.388
Male sex (vs female)	0.491	0.401	1.634 (0.744–3.586)	0.221
Pre-hospital ADL: total assistance	0.306	0.405	1.358 (0.614–3.004)	0.449
Pre-hospital cognitive impairment	0.714	0.520	2.043 (0.737–5.665)	0.170
Serum albumin, per 1-g/dL increase	−0.974	0.431	0.377 (0.162–0.879)	0.024

Variable	Coefficient β	SE	OR (95% CI)	P value
A-DROP severe/very severe	0.652	0.449	1.920 (0.796–4.632)	0.147

N=126; outcome events=70; parameters excluding intercept=6; log likelihood=−77.469; AIC=168.938; McFadden R^2=0.105; overall model likelihood-ratio P=0.006.

Table S1B. Full standard logistic regression results: Model 2

Variable	Coefficient β	SE	OR (95% CI)	P value
Intercept	0.309	2.693	1.362 (0.007–267.253)	0.909
Age, per 1-year increase	0.022	0.027	1.023 (0.970–1.079)	0.407
Male sex (vs female)	0.495	0.403	1.640 (0.744–3.617)	0.220
Pre-hospital ADL: total assistance	0.348	0.417	1.416 (0.626–3.203)	0.404
Pre-hospital cognitive impairment	0.750	0.536	2.116 (0.740–6.054)	0.162
Serum albumin, per 1-g/dL increase	−1.007	0.437	0.365 (0.155–0.860)	0.021
A-DROP severe/very severe	0.777	0.629	2.174 (0.634–7.460)	0.217
Antimicrobial pattern: overtreatment	−0.238	0.675	0.788 (0.210–2.962)	0.725
Antimicrobial pattern: undertreatment	−0.332	0.699	0.718 (0.182–2.826)	0.635
Antimicrobial pattern: regimen not specifically listed	−0.418	0.517	0.659 (0.239–1.814)	0.419

N=126; outcome events=70; parameters excluding intercept=9; log likelihood=−77.089; AIC=174.179; McFadden R^2=0.109; overall model likelihood-ratio P=0.026; global likelihood-ratio P for antimicrobial therapy pattern=0.859.

Table S1C. Full standard logistic regression results: Model 3

Variable	Coefficient β	SE	OR (95% CI)	P value
Intercept	−0.902	2.713	0.406 (0.002–82.748)	0.740
Age, per 1-year increase	0.029	0.027	1.029 (0.976–1.085)	0.287
Male sex (vs female)	0.491	0.410	1.634 (0.732–3.648)	0.231
Pre-hospital ADL: total assistance	0.380	0.418	1.463 (0.645–3.317)	0.363
Pre-hospital cognitive impairment	0.771	0.531	2.162 (0.763–6.126)	0.147
Serum albumin, per 1-g/dL increase	−0.898	0.445	0.407 (0.170–0.974)	0.044
A-DROP severe/very severe	0.476	0.465	1.609 (0.647–4.004)	0.306
Initial treatment failure within 7 days	1.193	0.562	3.296 (1.095–9.923)	0.034

N=126; outcome events=70; parameters excluding intercept=7; log likelihood=−74.962; AIC=165.925; McFadden R^2=0.134; overall model likelihood-ratio P=0.002.

Sensitivity analyses

Table S2A. Model 3 using a modified definition of initial treatment failure that excluded death as a qualifying criterion

Variable	OR	95% CI	P value
Serum albumin, per 1-g/dL increase	0.401	0.168–0.958	0.040
Modified initial treatment failure within 7 days	3.177	1.045–9.663	0.042

All 126 patients were retained, but death itself was removed from the definition of initial treatment failure. Model 3 included the same covariates as the primary analysis. N=126; outcome events=70. The direction, magnitude, and statistical interpretation of the principal associations were materially similar to those of the primary Model 3 analysis.

Table S2B. Model 3 after excluding the patient who died within 7 days

Variable	Coefficient β	SE	OR (95% CI)	P value
Intercept	−0.881	2.708	0.414 (0.002–83.610)	0.745
Age, per 1-year increase	0.028	0.027	1.029 (0.976–1.084)	0.294
Male sex (vs female)	0.480	0.410	1.616 (0.724–3.611)	0.242
Pre-hospital ADL: total assistance	0.372	0.418	1.451 (0.640–3.289)	0.373
Pre-hospital cognitive impairment	0.765	0.531	2.149 (0.760–6.080)	0.149
Serum albumin, per 1-g/dL increase	−0.889	0.444	0.411 (0.172–0.982)	0.045
A-DROP severe/very severe	0.486	0.465	1.626 (0.654–4.047)	0.296
Initial treatment failure within 7 days	1.161	0.566	3.192 (1.052–9.683)	0.040

N=125; outcome events=69; log likelihood=−74.849; AIC=165.697; McFadden R^2=0.129; overall model likelihood-ratio P=0.002.

Two patients who died within 48 hours of admission were excluded from the primary analytic cohort (N=126) according to the eligibility criteria of the submitted manuscript. To assess the robustness of the primary findings to this exclusion, all models were repeated in the full cohort of 128 patients, including these two early deaths.

Table S3. Sensitivity analysis including the two patients who died within 48 hours of admission (N=128)

Estimate	N=126 (primary, 48-h deaths excluded)	N=128 (sensitivity, 48-h deaths included)
Model 1 — serum albumin, OR (95% CI); P	0.377 (0.162–0.879); 0.024	0.368 (0.158–0.859); 0.021
Model 2 — serum albumin, OR (95% CI); P	0.365 (0.155–0.860); 0.021	0.356 (0.151–0.839); 0.018
Model 2 — antimicrobial therapy pattern, global LR P	0.859	0.838
Model 2 (Firth) — antimicrobial therapy pattern, PLRT P	0.883	0.870
Model 3 — serum albumin, OR (95% CI); P	0.407 (0.170–0.974); 0.044	0.403 (0.168–0.966); 0.042
Model 3 — initial treatment failure, OR (95% CI); P	3.296 (1.095–9.923); 0.034	3.431 (1.146–10.272); 0.028

Inclusion of the two early deaths did not attenuate any of the principal associations; the estimate for initial treatment failure was marginally strengthened (OR 3.296 to 3.431), and all associations retained the same direction and statistical significance as in the primary analysis. The absence of a statistically significant association between antimicrobial therapy pattern and outcome was also unchanged. These findings indicate that the direction, magnitude, and statistical interpretation of the principal associations were not materially sensitive to the exclusion of the two patients who died within 48 hours of admission. All values in this table were computed to three decimal places directly from the analytic dataset.

Table S4. Firth bias-reduced logistic regression for Model 2

Variable	Penalized coefficient β	OR (95% profile CI)	PLRT P value
Age, per 1-year increase	0.020	1.020 (0.970–1.075)	0.435
Male sex (vs female)	0.445	1.560 (0.738–3.381)	0.245
Pre-hospital ADL: total assistance	0.322	1.380 (0.629–3.030)	0.421
Pre-hospital cognitive impairment	0.675	1.964 (0.731–5.492)	0.182
Serum albumin, per 1-g/dL increase	−0.912	0.402 (0.171–0.884)	0.023
A-DROP severe/very severe	0.693	2.000 (0.637–6.791)	0.238
Antimicrobial pattern: overtreatment	−0.211	0.809 (0.222–2.837)	0.741
Antimicrobial pattern: undertreatment	−0.283	0.754 (0.198–2.758)	0.670
Antimicrobial pattern: regimen not specifically listed	−0.375	0.687 (0.256–1.809)	0.447

Global profile penalized likelihood-ratio P for the antimicrobial therapy pattern: 0.883. The conclusion was consistent with the standard likelihood-ratio test.

Table S5. Alternative adjustment sets

Analysis	N	Serum albumin OR (95% CI); P	A-DROP or age OR (95% CI); P	Initial treatment failure OR (95% CI); P	Residence OR (95% CI); P
Model 1 excluding age	126	0.384 (0.166–0.891); 0.026	A-DROP: 2.204 (0.964–5.035); 0.061	—	—
Model 3 excluding age	126	0.415 (0.175–0.986); 0.046	A-DROP: 1.938 (0.831–4.519); 0.126	3.095 (1.049–9.131); 0.041	—
Model 1 excluding A-DROP	126	0.360 (0.156–0.830); 0.017	Age: 1.037 (0.988–1.088); 0.141	—	—
Model 3 excluding A-DROP	126	0.396 (0.167–0.941); 0.036	Age: 1.039 (0.990–1.092); 0.121	3.628 (1.224–10.755); 0.020	—
Model 1 plus pre-hospital residence	126	0.347 (0.147–0.824); 0.016	A-DROP: 1.708 (0.690–4.230); 0.247	—	0.588 (0.216–1.599); 0.298
Model 3 plus pre-hospital residence	126	0.376 (0.154–0.917); 0.031	A-DROP: 1.460 (0.574–3.713); 0.426	3.234 (1.077–9.707); 0.036	0.607 (0.221–1.670); 0.334

Across alternative specifications, the directions and approximate magnitudes of the associations for serum albumin and initial treatment failure were materially unchanged.

Drug-resistant pathogen risk was not entered as an independent covariate in the primary models because the number of risk factors required for its classification depended on A-DROP severity, its criteria included hypoalbuminemia, and it was also used to define the initial empirical antimicrobial therapy pattern. Drug-resistant pathogen risk showed substantial overlap with A-DROP severity: 38 of 41 patients (92.7%) with severe or very severe A-DROP were classified as having drug-resistant pathogen risk, compared with 7 of 85 patients (8.2%) with mild or moderate A-DROP. To assess whether this omission materially affected the principal estimates, exploratory models additionally adjusting for drug-resistant pathogen risk were fitted for Models 1 and 3.

Table S6. Exploratory models with additional adjustment for drug-resistant pathogen risk

Estimate	Without drug-resistant pathogen risk	With drug-resistant pathogen risk added
Model 1 — serum albumin, OR (95% CI); P	0.377 (0.162–0.879); 0.024	0.380 (0.160–0.904); 0.029
Model 1 — drug-resistant pathogen risk, OR (95% CI); P	—	1.057 (0.255–4.373); 0.940
Model 3 — serum albumin, OR (95% CI); P	0.407 (0.170–0.974); 0.044	0.420 (0.173–1.023); 0.056
Model 3 — initial treatment failure, OR (95% CI); P	3.296 (1.095–9.923); 0.034	3.360 (1.107–10.194); 0.032
Model 3 — drug-resistant pathogen risk, OR (95% CI); P	—	1.270 (0.290–5.562); 0.751

In both models, drug-resistant pathogen risk itself was not statistically associated with non-return, and the estimated associations of serum albumin and initial treatment failure were materially similar to the primary models, with the association for serum albumin in Model 3 attenuating slightly (P=.056). An interaction between drug-resistant pathogen risk and the four-category antimicrobial therapy pattern was not estimated because the exposure classification was partly defined by that risk variable and several cross-classified groups were too small for reliable interaction estimates (e.g., only 3 patients with mild or moderate A-DROP and drug-resistant pathogen risk).

To assess whether the primary associations were consistent within the oldest segment of the cohort, Models 1 and 3 were repeated restricted to patients aged 80 years or older (N=107, 62 outcome events).

Table S7. Sensitivity analysis restricted to patients aged 80 years or older

Estimate	Full cohort (N=126)	Age ≥80 years (N=107)
Model 1 — serum albumin, OR (95% CI); P	0.377 (0.162–0.879); 0.024	0.375 (0.153–0.920); 0.032
Model 3 — serum albumin, OR (95% CI); P	0.407 (0.170–0.974); 0.044	0.408 (0.161–1.033); 0.059
Model 3 — initial treatment failure, OR (95% CI); P	3.296 (1.095–9.923); 0.034	3.733 (1.088–12.803); 0.036

The direction and approximate magnitude of the associations were generally consistent with those in the full cohort.

Table S8A. Exploratory analysis restricted to 30-day survivors: Model 1

Variable	OR	95% CI	P value
Age, per 1-year increase	1.02	0.96–1.07	0.571
Male sex (vs female)	1.45	0.66–3.22	0.357
Pre-hospital ADL: total assistance	1.25	0.56–2.83	0.585
Pre-hospital cognitive impairment	2.22	0.77–6.38	0.138
Serum albumin, per 1-g/dL increase	0.44	0.18–1.05	0.065
A-DROP severe/very severe	2.10	0.85–5.18	0.105

Table S8B. Exploratory analysis restricted to 30-day survivors: Model 3

Variable	OR	95% CI	P value
Age, per 1-year increase	1.02	0.97–1.08	0.431
Male sex (vs female)	1.52	0.68–3.40	0.313
Pre-hospital ADL: total assistance	1.35	0.59–3.11	0.476
Pre-hospital cognitive impairment	2.25	0.78–6.52	0.136
Serum albumin, per 1-g/dL increase	0.45	0.19–1.09	0.078
A-DROP severe/very severe	1.79	0.71–4.52	0.219
Initial treatment failure within 7 days	2.68	0.86–8.35	0.089

N=119 after excluding seven patients who died within 30 days. The directions of the associations were unchanged, but estimates were attenuated and no longer statistically significant. This analysis is exploratory because conditioning on survival may introduce selection bias.

Model diagnostics

Table S9A. Multicollinearity, influence, and model-complexity diagnostics

Model	Events/parameter	Maximum VIF	Adjusted GVIF for therapy pattern	Maximum condition index	Maximum Cook's D	Maximum leverage	Maximum |DFBETAS|	Cook flags	Leverage flags	DFBETAS flags
Model 1	11.7	1.226	—	1.719	0.048	0.102	0.349	3	0	24
Model 2	7.8	2.260	1.145	2.895	0.064	0.155	0.528	3	0	49
Model 3	10.0	1.226	—	1.744	0.063	0.139	0.436	5	2	29

Screening thresholds were Cook's distance >4/N, leverage >2k/N (k includes the intercept), and |DFBETAS| >2/sqrt(N). Some observations exceeded conservative screening thresholds, but no extreme influence values were identified. The maximum condition index was <3 in all models.

Table S9B. Leave-one-out robustness of key estimates

Model	Term	Full-data OR	Full-data P	LOO OR range	LOO P range	P<0.05 count	Valid fits	Interpretation
Model 1	Serum albumin	0.377	0.024	0.330–0.416	0.013–0.045	126	126	Direction unchanged in all fits
Model 2	Serum albumin	0.365	0.021	0.310–0.403	0.011–0.040	126	126	Direction unchanged in all fits
Model 3	Serum albumin	0.407	0.044	0.351–0.449	0.024–0.076	89	126	Direction unchanged; significance was borderline in some fits

Model	Term	Full-data OR	Full-data P	LOO OR range	LOO P range	P<0.05 count	Valid fits	Interpretation
Model 3	Initial treatment failure	3.296	0.034	2.926–4.313	0.016–0.057	123	126	Direction and magnitude were stable

Table S10. Restricted cubic spline tests for nonlinearity

Variable	Model	Knots (10th, 50th, 90th percentiles)	LRT df	P for nonlinearity
Serum albumin	Model 1	2.5, 3.1, 3.7 g/dL	1	0.459
Serum albumin	Model 3	2.5, 3.1, 3.7 g/dL	1	0.456
Age	Model 1	76.5, 89.0, 96.5 years	1	0.204
Age	Model 3	76.5, 89.0, 96.5 years	1	0.220

No evidence of departure from linearity was detected for serum albumin or age in Models 1 or 3.

Spline Figures

Figure S1. Restricted cubic spline assessment of serum albumin

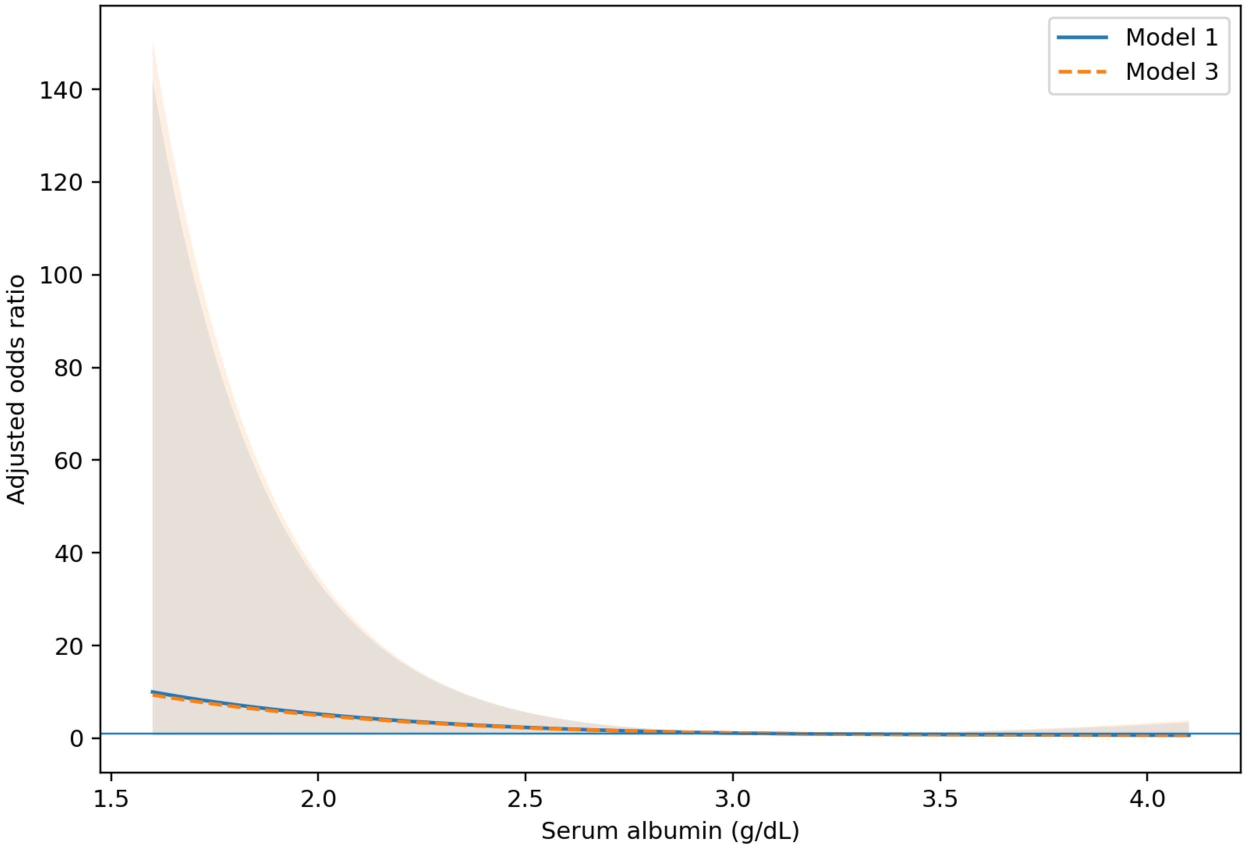

Adjusted odds ratios are shown relative to the median serum albumin value (3.1 g/dL). The curves were estimated from Models 1 and 3 using knots at 2.5, 3.1, and 3.7 g/dL.

Figure S2. Restricted cubic spline assessment of age

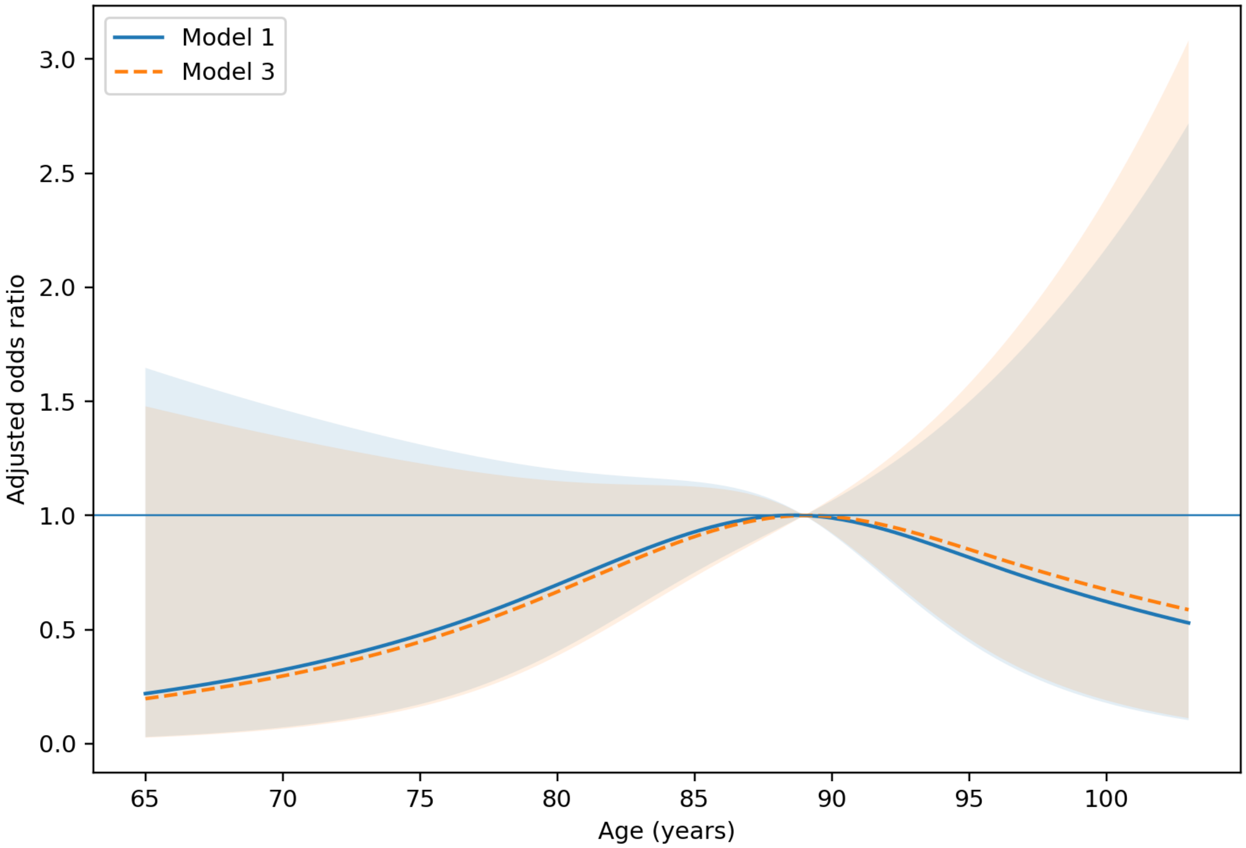

Adjusted odds ratios are shown relative to the median age (89 years). The curves were estimated from Models 1 and 3 using knots at 76.5, 89.0, and 96.5 years.

Summary of additional analyses. Across the sensitivity analyses, alternative adjustment sets, and diagnostic assessments, the directions and approximate magnitudes of the principal associations were generally consistent with those of the primary analyses. However, in the exploratory analysis restricted to 30-day survivors, the associations were attenuated and no longer statistically significant. These findings support the overall consistency of the primary results while also indicating limitations in statistical precision associated with the modest sample size.
